# The Integrated Health Monitor COVID-19: A Protocol for a Comprehensive Assessment of the Short- and Long-Term Health Impact of the Pandemic in the Netherlands

**DOI:** 10.3390/mps6060117

**Published:** 2023-12-02

**Authors:** Anouk van Duinkerken, Mark Bosmans, Christos Baliatsas, Nannah Tak, Anne Meerdink, Noortje Jansen, Marjonneke de Vetten-Mc Mahon, Elske Marra, Michel Dückers

**Affiliations:** 1 Nivel (Netherlands Institute for Health Services Research), 3513CR Utrecht, The Netherlandsm.duckers@nivel.nl (M.D.); 2 Department of Behavioral and Social Sciences, University of Groningen, 9712TS Groningen, The Netherlands; 3 GGD GHOR Nederland (Overarching Organization of the Municipal Health Services), 3524JS Utrecht, The Netherlands; 4 ARQ National Psychotrauma Centre, 1110AE Diemen, The Netherlands; 5 RIVM (National Institute for Public Health and the Environment), 3720BA Utrecht, The Netherlands

**Keywords:** COVID-19, coronavirus, pandemic, public health, pandemic, longitudinal, mental health, lockdown

## Abstract

Background: The global COVID-19 pandemic has profoundly affected public health. Directly, the pandemic resulted in over 6.6 million deaths, numerous hospitalizations, and widespread illness. The pandemic has also affected health indirectly through government-imposed protective measures, causing decline in mental well-being and increasing social isolation. Unlike previous disasters or crises, the pandemic’s worldwide and enduring impact necessitates a unique research approach. The Network for Health Research in Disasters in the Netherlands responded by initiating a longitudinal, extensive research project called the Integrated Health Monitor COVID-19. The Integrated Health Monitor COVID-19 explores both the direct and indirect health effects of the pandemic at the population level. Methods: The Integrated Health Monitor COVID-19 employs a dual-pronged monitoring strategy alongside an annual literature review. This strategy comprises short-cycle monitoring (conducted quarterly) and long-cycle monitoring (conducted once every one or two years). This comprehensive approach enables the evaluation of health trends during the pandemic, facilitating comparisons with pre-pandemic levels and identification of risk and protective factors. Both monitoring methods incorporate data from surveys and general practice registries. The integration of annual literature reviews with these measurements enables iterative research, while dialogues on policy and practice improvements enhance the knowledge-to-action process. Discussion: Much of the existing knowledge about the potential impact of the COVID-19 pandemic is derived from research on sudden-onset disasters limited to specific geographical areas. This study is anticipated to provide valuable fresh insights into the evolving dynamics of population health and specific vulnerabilities within the ongoing pandemic context.

## 1. Introduction

In March 2020, COVID-19 was declared a global pandemic by the World Health Organization [1]. With over 630 million infections and more than 6.6 million deaths related to COVID-19 globally as of November 2022, this pandemic is a prime example of a health crisis [2]. Its health effects can exceed communities’ capabilities to address and deal with the consequences [3]. Consequently, governments implemented various protective measures, such as lockdowns, remote work and study arrangements, and social distancing, with the aim of reducing virus transmission and alleviating the strain on healthcare systems caused by the health effects of the virus [4].

Conflicting interests played a part in the decision-making process during this crisis. Direct negative effects of the virus were weighed against the indirect negative (health) effects of the countermeasures taken. These indirect effects frequently resulted from delays in accessing healthcare services, heightened feelings of isolation and loneliness, alterations in lifestyle, and financial hardships such as income loss or job insecurity attributable to the pandemic [5,6,7,8,9]. Importantly, these direct and indirect effects do not affect everyone equally. The pandemic is recognized as a syndemic, indicating that the severity of the COVID-19 pandemic has been magnified by existing inequalities in chronic diseases and social determinants of health [10], and in turn, these inequalities have been reinforced through the pandemic [11]. As a result, health differences between vulnerable groups and the rest of the population have increased. Furthermore, more people have become part of vulnerable populations due to the pandemic, for instance, as a result of long-COVID symptoms or job loss, thereby exacerbating the problem.

According to Malilay et al. (2014), “the application of epidemiology in disaster settings, also known as disaster epidemiology, can provide actionable information for use by policymakers, planners, incident commanders, decision-makers, and affected community members” [12] (p. 2092). From this perspective, disaster epidemiology, disaster health research or other names given to the process of researching the mental and physical health effects of a disaster, crisis, or incident and the need for assistance among those affected is a means to an end, a source of actionable information. The outcomes of this type of research are important for optimizing care and assistance for the affected population. Traditionally, health research after a disaster or crisis is mostly limited to local and/or acute disasters, focusing on the direct consequences for those affected [13]. However, with the COVID-19 pandemic being classified as a creeping crisis, it is uncertain, even doubtful, to what extent it is comparable to other crises. The COVID-19 crisis is slower burning: it has a long incubation time and persistent consequences long after its peaks. Furthermore, it involves an element of uncertainty surrounding the end of this crisis. These are all characteristics of a creeping crisis [14]. The nature of this crisis makes it exceptional. Such as the longevity, complexity, impact on all aspects of the lives, health, and well-being of citizens, together with its global magnitude. As a result, its health effects may differ from those of other disasters; thus, a different research approach is needed.

As the COVID-19 pandemic deviates from traditional disasters (and thus its research protocols), it requires a national approach that enables longitudinal research encompassing the full range of possible health effects. From this standpoint, the Integrated Health Monitor COVID-19 emerged as an integral, overarching research structure. It brings together different organizations in a supraregional approach with national coordination. Leveraging existing research and monitoring infrastructure, this initiative has been scaled up and extended to align with the unique research requirements of this ongoing crisis. This structural framework facilitates comparisons over time and across regions, enabling the identification of risk groups.

The focus of the current study is on the Netherlands, a high-income country with approximately 17.8 million inhabitants [15] that, like other countries, was confronted with deaths, hospitalizations, and a crisis approach that focused heavily on protecting acute healthcare capacity at the risk of societal consequences [16].

### 1.1. Aims

The primary objective of the monitor is to provide valuable information on the physical and mental health effects of the COVID-19 pandemic and its related protective countermeasures on the Dutch population. These health effects include changes in physical health, mental health, and (social) well-being. Where possible, a distinction is made between the direct health effects of the virus, the indirect health effects of protective measures, and the overall impact of the pandemic. The subgoals of the monitor are as follows:

1. To gain insight into the physical health impact of the COVID-19 pandemic at the population level;
2. To gain insight into the mental health impact of the COVID-19 pandemic at the population level;
3. To gain insight into the impact of the COVID-19 pandemic on social well-being at the population level;
4. To gain insight into the risk and protective factors of the direct and indirect health effects of the COVID-19 pandemic and to identify potentially vulnerable groups;
5. To ensure that this knowledge reaches and can be used by national, regional, and local policymakers (actionable information).

**Figure 1:**
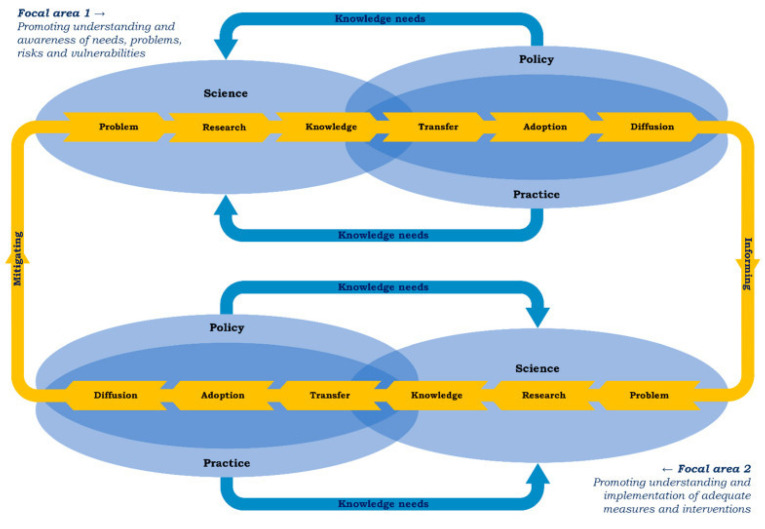
Two focal areas describing the circular knowledge-to-action process.

The process of the fifth subgoal is depicted in Figure 1, which shows two focal areas within a series of steps that influence each other [17]. The Integrated Health Monitor COVID-19 is shaped along a dialectic process in which the focus is determined with national and regional stakeholders from policy practice and science to plan, collect, and analyze data and disseminate findings (the upper part of the model). It also facilitates the start of a process to formulate solutions to relevant needs, problems, and risks with similar stakeholders at the national and regional level (the lower part of the model). Thus, the Integrated Health Monitor COVID-19 seeks to produce reliable information about the health impact of the pandemic on policy and practice.

### 1.2. Expected Results

The crisis approach in the Netherlands placed a strong emphasis on protecting acute healthcare capacity at the risk of societal consequences. This approach resulted in multiple phases of social distancing measures and lockdowns. This could lead to heightened stress and mental health challenges due to factors such as social isolation, economic uncertainties, and disruptions in daily life, potentially affecting the younger generations the most. Moreover, there are potential implications for physical health, stemming both directly from COVID-19 infections and indirectly from changes in healthcare access and utilization. This aspect could particularly affect the older generations. The complex interplay of these factors underscores the need for a comprehensive understanding of the multifaceted impacts of the crisis approach on both mental and physical well-being across different age groups.

## 2. Materials and Methods

### 2.1. Network for Health Research in Disasters and the Integrated Health Monitor COVID-19

The Network for Health Research in Disasters is a coordinating body for the planning and implementation of disaster health research during the COVID-19 pandemic. Key actors in this network include the National Institute for Public Health and the Environment (RIVM), the Municipal Health Services (GGDs) in collaboration with GGD GHOR (which is the overarching branch organization of the 25 GGDs), the Netherlands Institute for Health Services Research (Nivel), and the ARQ National Psychotrauma Centre.

**Figure 2:**
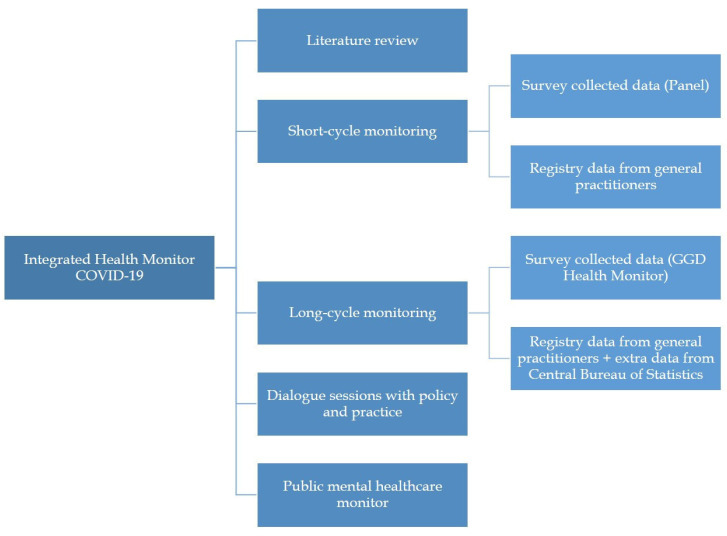
An overview of the components of the Integrated Health Monitor COVID-19.

This consortium designed a national monitoring program to track the effects of the COVID-19 pandemic on health. It received funding for five years, from 2021 to 2025, to ensure regular updates. The monitoring process employs an iterative approach of posing hypotheses, testing them by integrating existing data with newly gathered and registry data, and then integrating these insights into the next round of data collection. Different methodologies and data sources are brought together in this monitor, consisting of three main components: short-cycle monitoring, long-cycle monitoring, and literature reviews of international and Dutch studies. Each of these components has distinct objectives, along with separate data collection and analysis. Short-cycle monitoring involves periodic updates to track trends, and long-cycle monitoring involves longitudinal analyses of data on health and a broad variety of health determinants to analyze patterns and vulnerable groups. The literature reviews provide an overview of existing research on the health effects of the pandemic and can inform decisions made with short- and long-cycle monitoring. Addressing the fifth subgoal, special attention is given to specific socially vulnerable groups in the public mental healthcare (PMHC) monitor. Finally, to enhance the dissemination of knowledge from science to application in policy and practice, dialogue sessions are organized with relevant stakeholders. Figure 2 illustrates the different components of the monitor, each of which will be detailed further below.

The outcome parameters used in the Integrated Health Monitor COVID-19 are based on earlier health disaster research [18,19,20,21,22,23,24], such as (posttraumatic) stress and suicidal thoughts. Other outcome parameters included are those that were already present in the existing research structures for comparison with the pre-pandemic situation. For example, loneliness, well-being, and lifestyle factors. Additional parameters that are specifically relevant to the situational context of the COVID-19 pandemic are also included, such as possible symptoms of the virus or long COVID and potentially delayed care (due to protective measures). A complete overview of these abovementioned health outcomes is included in the Supplementary Tables.

A number of youth councilors, municipalities, and aid organizations stated In a letter of concern to the government that youth, especially vulnerable youth, have been severely impacted by the pandemic. They mention that the COVID-19 crisis brings significant risks for youth, particularly in the areas of education, employment, mental health, and personal income. Moreover, they will face the long-term economic and social consequences of the crisis. Reflecting these concerns and at the request of the subsidizers of the study, the first year of the Integrated Health Monitor COVID-19 specifically focuses on youth (aged 0 to 25) and the distinct vulnerable groups within this population.

### 2.2. Literature Review

Annually, a systematic literature review is conducted on the health effects of the COVID-19 pandemic. This review delves into six key domains: (1) physical health effects, (2) effects on healthcare needs and usage, (3) mental health effects, (4) social effects, (5) additional indirect effects, and (6) risk and protective factors. These domains are based on previous disaster health research and cover both the direct (through infection) and indirect (through measures taken to reduce transmission) effects of COVID-19. The primary objectives of these literature reviews are twofold: firstly, to compile a comprehensive overview of the pandemic’s effects on the general population and vulnerable groups; secondly, to enhance monitoring efforts by identifying knowledge gaps and providing input for further, in-depth research, thereby informing other aspects of the monitoring process. The yearly repetition of the literature review enables the assessment of the impact of the pandemic over time. In the first year (2021), the systematic literature review focused on youth and young adults (up to 25 years old). The second literature review is an umbrella review to cover the large number of studies published on the broad topics mentioned for all populations. Following this, subsequent reviews focus on more specific themes. Examples of mental health and social outcomes include shifts in mental health symptoms and disorders, suicidality, general well-being, social support, and loneliness. Other themes include delayed healthcare and work absenteeism. The selection of these themes is based on the findings of previously executed components of the monitor. Examples of the literature reviews’ findings are presented in Box 1.

Box 1 **Example of findings: literature reviews.**

     Results of the first two literature reviews show that the pandemic impacted the physical, mental, and social health of the population. This impact is not only due to the direct effect of an infection with the virus but also due to the indirect effect the public health measures and the experienced threat of the pandemic. Main effects are as follows:

- The pandemic and associated measures had a persistent negative impact on mental health: especially among adolescents and young adults, depression, anxiety, and loneliness increased;
- Postponed healthcare and late diagnoses of certain diseases had a big impact on health, particularly for people who were already ill or fell ill during the pandemic. This has led to an irreparable loss of healthy years of life;
- Over half those who contracted COVID-19 still had physical complaints, such as fatigue, three months after being infected;
- Many people working in healthcare suffer from physical and mental exhaustion due to the pandemic.

     A final important finding was that vulnerable groups—such as people with a low income and specific age groups—are impacted most strongly by the pandemic, with plausible widening of health gaps as a result.

     The detailed descriptions of these findings are available in two publicly accessible publications in Dutch [25,26].

For the international overview, the primary search encompasses three international databases: PubMed (including all records from MEDLINE), PsycINFO, and Embase. The search strategy and screening are carried out independently for each domain. Screening is conducted in three phases: title screening, abstract screening, and full-text screening. The inclusion criteria mandate that articles (1) be published in peer-reviewed journals, (2) be longitudinal studies with multiple measurements (including registry studies), (3) focus on the context of the COVID-19 pandemic (including the impact of restrictive measures), (4) have health problems/conditions or health status scores as the dependent variable, (5) be available in English, and (6) encompass all populations (e.g., general population, COVID-19 patients, healthcare staff). The criteria exclude studies that focus on pathogenesis and virologic mechanisms or animal studies, studies with a different design (e.g., cross-sectional or experimental), editorials, anecdotal descriptions, letters, case reports, and gray literature.

Following screening, all included studies undergo a quality assessment concerning methodological rigor using validated instruments. In the first year, a combination of several validated instruments (the AXIS Quality Assessment tool [27], the Mixed Methods Appraisal Tool [28], and the Critical Appraisal Programme [29]) is used for quality assessment. After consensus on the included items and pilot testing within the research team, we chose a combined instrument consisting of items that cover fundamental aspects of methodological quality and risk of bias explicitly relevant to observational studies (cohort, longitudinal, case–control and cross-sectional). Moreover, given the substantial volume of data/studies assessed by different reviewers/projects, we aimed to include items/questions that were clearly stated and easily interpretable. In the second year, an adapted version of AMSTAR 2 [30] is utilized to evaluate the quality of the reviews included in the umbrella review. AMSTAR is recognized as one of the most extensively utilized tools for assessing the quality of systematic literature reviews, comprehensively examining various facets of their conduct and reporting. For both screening and quality assessment, interrater reliability is assessed by independent quality scoring of 30% of the included studies, whereby differences in rating are resolved through discussion among the involved screeners.

Additionally, an annual scoping review of Dutch research on the effects of the COVID-19 pandemic is undertaken. Special attention is dedicated to national research due to its relevance in the national context for Dutch policymakers. This review includes literature reviews and large-scale longitudinal studies from relevant research institutes, such as research inventories from RIVM and reports from different Dutch research institutes that are relevant to the population or topic at hand. These inclusion criteria are less stringent than those for the international literature review, signifying the inclusion of not only studies with multiple measurements published in scientific journals but also reports and inventories.

### 2.3. Short-Cycle Monitoring

Short-cycle monitoring provides insight into physical and mental health problems in the general population at regular intervals throughout the year, with four assessments conducted annually. This approach allows for tracking trends over time, facilitating the identification and signaling of ‘real-time’ changes in health patterns. Consequently, it becomes possible to respond promptly to sudden fluctuations in health conditions through appropriate policy measures. Moreover, quarterly updates allow for the consideration of seasonal changes, both in terms of airborne diseases and mental status.

The short-cycle monitor uses two data sources: a panel study and data from general practitioner registries. Both data sources are analyzed independently, yet comparisons are made between them where possible to examine how the patterns of health problems in self-reported surveys correspond with the patterns of health problems and diagnoses in primary care. Box 2 provides examples of findings from both data sources in short-cycle monitoring.

Box 2 **Example of findings: short-cycle monitoring.**

     Based on quarterly panel surveys among youth and young adults (12–25) since September 2021, it was concluded that the mental health of this population deteriorated after the last severe lockdown period in the Netherlands during the winter of 2021/2022. The first measurement of 2022, for instance, indicated an increase in suicide ideation experienced by participants during the last three months from 8.5% (December 2021) to 16.8% (March 2022). Later measurements gave little sign of recovery. A year later, the suicide ideation in youth and young adults was 14.1% (March 2023), remaining above the pre-lockdown level.

     A multivariate analysis of the survey data confirmed that suicide ideation was significantly higher among youth and young adults with mental health problems and emotional loneliness, i.e., a lack of an intimate connection with others, regardless of the number of contacts. Moreover, survey participants reported being too busy, experiencing higher levels of stress, and having insufficient time to relax. Youth and young adults are worried about the future. They express concerns about their financial situation, the housing market, and experience high levels of performance pressure.

     The panel survey data and information on the analysis are presented on the RIVM’s Integrated Health Monitor COVID-19 website [31].

The longitudinal development of mental health, including suicidality, in the weekly general practitioner registry data follows a similar pattern. General practitioners registered a 24% higher prevalence of suicidality consults per 100,000 patients in the first quarter of 2023 [32].

#### 2.3.1. Panel Surveys

The first component of short-cycle monitoring comprises panel surveys conducted every three months. These online questionnaires collect information regarding the health status and support needs of the Dutch population, with the possibility of comparing groups based on their risk profile (i.e., particular combinations of health determinants). Panel studies provide insight into trends in health effects over time and across subpopulations that are at increased risk of experiencing the negative effects of the pandemic. The frequent data collection ensures that the data is up to date and offers information about the present situation while enabling the tracking of changes in health and support needs over time. The findings are reported on publicly available dashboards (e.g., websites or online factsheets) and discussed with key stakeholders on the national and regional levels as part of the dialogue shown in Figure 1. Thus, signals of health deterioration and increased support needs can be identified and responded to quickly by local, regional, and national policy and practice institutions.

The survey is conducted by I&O Research on behalf of Network for Health Research in Disasters. Panel members for the I&O Research Panel are selected through random sampling and actively recruited by the organization. Typically, they employ sampling methods from (municipal) population registers or extensive address databases. The organization deliberately chooses not to allow self-registration, as it may result in a bias where only individuals interested in research (professional respondents) sign up. Therefore, new panel members are actively approached and asked if they want to join the panel. The organization already possesses significant background information on panel members, enabling them to draw representative samples or target specific demographics for research purposes. I&O research utilizes a loyalty program to incentivize panel members to participate and enhance their engagement with the panel. Panel members receive reward points for completing questionnaires, with the number of points depending on the length and complexity of the questionnaire. These points can be redeemed for gift cards or donations to charitable organizations. Through the loyalty program, the organization ensures a balanced participation from panel members driven by interest and engagement in research as well as those participating for personal benefits.

The study population of the panel survey is people aged 12 and older. This study population is divided into two subpopulations: youth and young adults (ages 12 to 25) and adults (ages 26 and older). People are invited and reminded to participate via email. Informed consent was obtained prior to participation. After data collection, the data are weighted based on gender, age, level of education, and region.

The outcome measures are related to general health and well-being (mental and physical), support needs, and the perceived impact of the COVID-19 crisis. Themes explored in the surveys encompass perceived social support, stress, physical health, and the perceived impact of the COVID-19 pandemic on one’s life. A combination of preselected health determinants is also included, covering social demographic characteristics, social context, exposure to pandemic-related risk factors, and life events. Where possible, validated constructs are used. For example, the PCL-5 is used to measure symptomology related to posttraumatic stress disorder (PTSD) [33]; the MHI-5 is used to measure psychological complaints [34]; a shortened version of the loneliness scale by de Jong-Gierveld is used to measure loneliness [35]; and a selection of “nonspecific symptoms”, covering mental and physical health items, is included based on the Symptoms and Perceptions tool (SaP) by Yzermans et al. [19]. These symptoms include common symptoms and complaints, which are often nonspecific, frequently exist in the population, and can have a psychosomatic component. A subset of these complaints have been identified as possible symptoms of long COVID [36], but they are not limited to those who had a COVID-19 infection. Where possible, questions are aligned with other components of the Integrated Health Monitor COVID-19, facilitating meaningful comparisons between the results from short-cycle and long-cycle monitoring (which is described in Section 2.4).

The data are presented cross-sectionally for each panel report and longitudinally after three reliable and consistent data points are collected. The data presented in the reports are mostly descriptive. Where possible, trend graphs are presented to identify patterns. Risk factors are analyzed for each outcome separately through a two-step process. First, random forest regressions are conducted to identify which risk factors have a substantial association with the outcome. Second, based on the outcomes of the random forest regression, appropriate multivariate/multivariable models are applied to quantify the strength of the associations between risk factors and the outcome.

#### 2.3.2. Monitoring Based on Weekly General Practitioner Registry Data

The second component of short-cycle monitoring is based on general practitioner registry data and encompasses four reports per year used to gain insight into the prevalence of a diverse range of diagnosed health problems and symptoms. In the Netherlands, the majority of residents are registered at a general practice located in close proximity to their place of residence, so the population listed in family practice can serve as the denominator in epidemiological studies [37]. Therefore, the general practice registered data provide a complete picture of the health characteristics of primary care patients in the Netherlands. An opt-out system is available for patients who choose not to have their data utilized.

This component uses data from a subset of the Nivel Primary Care Database (PCD), a substantial research infrastructure based on real-world data extracted from routine electronic health records (EHRs) of general practitioners and primary out-of-hours care services [38]. The subset used is the Nivel Surveillance data, which consist of approximately 390 general practices (including a network of more than 40 sentinel general practices) that participate in the PCD and provide routinely collected data on a weekly basis [39].

The general practice registers information on health problems, consultation rates, diagnoses, prescribed medication, and referrals in the EHRs. The registration system that is used follows the International Classification of Primary Care (ICPC) [40], which is the standard for coding and classifying complaints, symptoms, and conditions under a general practitioner’s care within the Netherlands. Additionally, the Anatomical Therapeutic Chemical (ATC) classification system categorizes medication based on the organ or system they affect [41]. Since these codes are used by all Dutch general practitioners, they generate nationally representative data on primary care.

The weekly prevalence of 20 relevant symptoms and complaints is documented for a continuous period of three months to regularly provide a representative real-time overview of the population’s health in the Netherlands. The selection of symptoms is based on an overview provided in the SaP instrument [19]. The symptoms and complaints included in the analyses and their corresponding registration codes can be found in the Supplementary Material. Suicidality, encompassing completed suicides, suicide attempts, and suicidal thoughts, is included as well, given its potential relevance due to the adverse effects of disasters and crises on mental health. In the case of the COVID-19 pandemic, these adverse effects can be related to lockdowns and social isolation.

The weekly prevalence of these aforementioned symptoms is defined as the number of people who consulted the general practitioner in a given week for certain symptoms or disorders divided by the total number of patients registered in the general practice. The data from the previous three months are compared to a similar period (the same weeks) in other years, both before and during the pandemic. Comparisons are made between men and women, age groups, and different Dutch provinces, they are presented in graphs as prevalence per 100,000 people.

### 2.4. Long-Cycle Monitoring

Within the framework of long-cycle monitoring, a substantial body of data is collected and analyzed (bi)annually to provide insight into trends, the development of health problems and care utilization, and relevant risk and protective factors. This approach also facilitates comparisons over time and across different regions. Long-cycle monitoring consists of two components: firstly, the GGD Public Health Monitor, which is conducted among three different age groups: youth (grades 2 and 4 of secondary school), young adults (ages 16 to 25), and adults and elderly people (ages 18 and older); and secondly, general practitioner registry data combined with data from the Dutch Central Bureau of Statistics. Examples of findings from the long-cycle monitoring are detailed in Box 3.

Box 3 **Example of findings: long-cycle monitoring.**

     According to findings from the GGD Public Health Monitor, specifically the Youth Monitor, second and fourth graders of secondary school are less happy than before the pandemic (compared to 2019). Experiences during the pandemic, such as quarantine, a coronavirus infection, and illness or death of a loved one can heavily impact their well-being. One-fifth of the students in the second and fourth grade reported having suicidal thoughts in the year prior to completing the questionnaire: 17% indicated experiencing such thoughts “occasionally” or “sometimes”, while nearly 5% reported experiencing them “(very) often”.

     These findings are presented in a news release available on the RIVM’s Integrated Health Monitor COVID-19 website, in Dutch [42].

     An initial analysis of general practitioner registry data reveals a decline in primary healthcare utilization in 2020 compared to 2019. As a result, most health complaints and problems are registered less frequently by general practitioners. However, health issues differ between potentially vulnerable groups based on risk factors:

- Women and individuals with a low income exhibit more psychological and anxiety-related complaints.
- Those with a migration background report more social problems.
- Patients with preexisting physical complaints experience less shortness of breath.
- Patients with preexisting psychological problems report more shortness of breath but fewer issues related to healthcare accessibility and social problems.

     The findings are elaborated in a publicly available report in Dutch [43].

#### 2.4.1. GGD Public Health Monitor

The GGD Public Health Monitor comprises questionnaire surveys developed and disseminated by municipal health services. It was established to keep track of the population’s public health and to inform health policymakers of relevant issues and developments. The survey covers topics such as lifestyle, (mental) health, substance use, potentially traumatic experiences, and life events during the pandemic. Originating as an existing survey structure, it intensified during the pandemic, incorporating additional topics concerning the effects of the COVID-19 pandemic in the questionnaire. Similar to the approach described earlier for short-cycle panel surveys, long-cycle instruments are based on validated instruments where possible. Special attention is dedicated to enhancing the comparability and overlap between the questionnaires for the different groups across years and across other components of the overarching Integrated Health Monitor COVID-19. After data collection, all data are weighted or stratified based on gender and age to ensure representability for the general population.

First, the Youth Public Health Monitor is conducted among children in the second and fourth grades of secondary school. Before the COVID-19 pandemic, it was conducted every four years. However, in response to the pandemic, its frequency was adjusted to every two years. All schools offering regular secondary education are invited to participate in the study through email or phone communication. Information about the study is included in newsletters and advertised on websites. Both parents and the children themselves can opt out of the study by contacting the school, without the obligation to provide a reason for refusal to participate. The Youth Public Health Monitor was conducted in 2015, 2019, and 2021.

Second, the Adults and Elderly Health Monitor is conducted among two target groups: adults (ages 18 to 64) and elderly people (ages 65 and older). Much like the Youth Health Monitor, the frequency was adjusted from once every four years to once every two years in response to the pandemic. The two target groups are sampled separately due to a difference in the expected response rate. This separation allows for independent presentations of the results. Random samples per target group are drawn from pools stratified by area. Participants are invited by letter to take part in the survey. The Adults and Elderly Health Monitor was conducted in 2012, 2016, 2020, and 2022.

Finally, a new health monitor was established for young adults to obtain better insight into the public health of this specific group during the pandemic. This age group was underrepresented in the existing monitoring structure and 17-year-olds were not included in any of the existing monitors yet. The Young Adults Health Monitor is administered among young adults ages 16 to 25, with participants primarily recruited through social media. This Health Monitor for this specific age group is motivated by two key factors. Firstly, the response rate from this demographic in the Adults and Elderly Health Monitor was suboptimal, prompting the introduction of the Young Adults Health Monitor as an additional initiative to engage this age cohort more effectively. Secondly, 17-year-olds were not encompassed in either the Youth Health Monitor or the Adults and Elderly Health Monitor, underscoring the necessity for a distinct monitoring approach. The first time this monitor was conducted is in 2022, and it will be repeated in 2024.

Data analysis primarily consists of descriptive statistics presented on national, regional, and local levels. Where possible, comparisons are drawn between other GGD Public Health Monitors (previous measurements and/or other populations). Furthermore, at the national level, possible risk and protective factors are identified by comparing outcomes between groups.

#### 2.4.2. General Practitioner Registry Data

The second component of long-cycle monitoring is based on general practitioner registry data combined with data from other sources, which are used to gain insight into the development of diagnosed health problems and health symptoms over time and to compare different potentially vulnerable groups. This component provides a means to understand trends and patterns and is conducted annually. The difference between short-cycle monitoring and long-cycle monitoring using general practitioner registry data lies in the latter’s capacity for more extensive, in-depth analyses by statistically testing differences between years (including years before the pandemic as a baseline/reference) and population groups in the occurrence of health problems while accounting for various potentially confounding factors. This approach aids in identifying groups that warrant special attention, thereby enhancing existing knowledge about the health implications of prolonged crises.

Similar to short-term monitoring, the data are obtained from the EHRs of general practices in the Nivel PCD, which contains data from approximately 10% of the Dutch population (±1.7 million citizens) from approximately 500 general practices [44]. Individual-level merging of the EHR dataset with data on outcomes (e.g., suicide prevalence) and individual and contextual characteristics, such as indicators of socioeconomic status and migration background (sourced from the Dutch Central Bureau of Statistics and GGD Public Health Monitors) is possible. A comprehensive overview of the outcomes from the different databases and the operationalization of the variables is included in the Supplementary Material.

For data analysis, longitudinal/panel data mixed-effects regression models are employed. Given the hierarchical structure of the data, with patients clustered in general practices, multilevel regression analyses are executed. These analyses primarily involve logistic regressions (for the prevalence of health complaints and diagnoses) and negative binomial regressions (for the number of consults/visits) with time as a predictor to test differences in years. In each model, background variables are added to function as control variables, namely, age, sex, socioeconomic background (income), migration background, and how long someone was registered to that general practice during the year. To assess the development of outcomes over time across different risk factors (age groups, gender, socioeconomic status, migration background, preexisting (mental) health problems, and preexisting social problems), an interaction variable (risk group*year) is generated and added to the model.

### 2.5. Dissemination and Implementation

Beyond gathering data about the health effects of the COVID-19 pandemic, the Integrated Health Monitor COVID-19 aims to translate these findings into suitable care and support through collaboration with national and local professionals, policymakers, and experts. To facilitate this process, a comprehensive network of active connections and relevant local stakeholders has been established. These stakeholders receive invitations to contribute to dissemination and implementation sessions and participate in focus groups. Throughout these sessions and groups, the research findings are translated into accessible and practical language, ensuring clarity and ease of utilization for stakeholders. The aim is to facilitate the absorption and practical implementation of the data.

### 2.6. Public Mental Healthcare Monitor

The Public Mental Healthcare (PMHC) monitor was established to target vulnerable groups typically overlooked in registry or survey research. The target group of the PMHC monitor is those who are socially excluded and possibly not self-sufficient, which primarily includes marginalized groups, such as homeless people, refugees, people with severe mental health problems, or people with problematic debts. The primary objective of this monitor is to gain insights into the impact of COVID-19 on those least visible but possibly most severely affected, providing guidance for policymaking concerning care and support needs. Methodologically, this monitor is based on a mixed-methods approach using secondary data. From a quantitative perspective, a yearly analysis of a set of indicators from registries, such as data from the police, mental health services, and emergency services, is analyzed. This is performed to create a general overview of the problems experienced by these vulnerable groups and how these issues developed during the pandemic compared to before the pandemic. From a qualitative perspective, existing interviews and focus groups with (representatives of) target groups are assessed and combined to elucidate the experiences and narratives of the people behind the quantitative data. The analysis of secondary data serves to identify knowledge gaps for future data collection within this monitoring framework. The process of data collection has not been specified yet but will undergo appropriate ethical approval steps when necessary.

## 3. Discussion

### 3.1. A Broad Health Monitor Based on Multiple Data Sources

The Integrated Health Monitor COVID-19, outlined in this article, aims to establish a comprehensive information base for the physical and mental health effects of the COVID-19 pandemic and to identify risk and protective factors for these effects. The development of the monitor stems from the wanting to overcome the limitations of cross-sectional studies. Understanding the far-reaching consequences of an ongoing crisis necessitates a study design that systematically tracks health dynamics over time. This longitudinal monitoring can contribute to disaster health research preparedness in the future and has the potential to strengthen the Dutch public health research infrastructure.

### 3.2. Implications for Policy and Practice

The results of the Integrated Health Monitor COVID-19 can be used to inform policy and practice. However, the translation from knowledge to action in policy and practice is challenging, especially within the decentralized care system in the Netherlands. Each region has its own stakeholders and executors with possibly conflicting views, interests, and priorities. This diversity is likely to influence the sense- and decision-making processes related to formulating solutions, as well as the implementation of such solutions, both at the local and national level. To enhance the effectiveness of translating knowledge to action, the monitor places a particular emphasis on this process by inviting these stakeholders into sessions to discuss and interpret the research results.

Social vulnerabilities can be reinforced due to crises such as the COVID-19 pandemic. It is crucial for policymakers to take into account that vulnerable groups may need more support to mitigate the negative effects of the pandemic. The monitor aims to provide insight into the risk and protective factors for these negative health effects to facilitate policymaking. Implementing preventive measures to mitigate these effects and enhance the health of both the overall population and specific vulnerable groups is imperative. Preventive measures entail broader efforts beyond only addressing the direct causes and effects of the pandemic but also creating an overall improvement in public health. Such a holistic approach can empower the population to navigate potential resurgences of the pandemic and cope with other future disasters and crises.

The applicability of the methodology outlined in this protocol to other countries is a matter of debate. The Integrated Health Monitor COVID-19 is not intended for direct replication in different (national) contexts, because this research protocol is tailored to the Dutch healthcare system where primary care functions as a gatekeeper and a systematic, standardized registry system is used. Despite not being directly replicable, the fundamental principles of longitudinal monitoring and upscaling existing research structures can be applied elsewhere. Instead of relying on applying identical tools, other regions can utilize large-scale, representative existing panels or longitudinal surveys, as well as other available data structures, to establish a baseline. Although the monitor’s outcome parameters are rooted in disaster health research rather than being exclusive to the COVID-19 pandemic, they can be adapted for diverse settings and offer guidance for future disaster health research practices in the Netherlands and elsewhere. To apply this monitoring structure elsewhere, it is important to adjust the outcome parameters to the relevance of the wider cultural and socioeconomic context of populations. At the country level, these characteristics have been confirmed to differ substantially. Several cross-national comparison studies point to a positive association between country wealth metrics and the prevalence of mental health outcomes in general [45,46,47,48] and in combination with exposure to disasters or other potentially traumatic events [49,50]. This highlights the importance of adapting to cultural and socioeconomic context.

### 3.3. Limitations and Strengths

A limitation of the monitor lies in the challenge of attributing observed health outcomes to specific factors. Distinguishing between the effects of a COVID-19 infection and the impacts of the protective countermeasures is intricate. Individuals do not exist in a vacuum in which the effects of only infection or countermeasures can be measured. It is important to clarify that the monitor does not claim to achieve this specific distinction, since its primary objective is to evaluate the overall health effects of the pandemic as a whole. Additionally, the longitudinal design of the monitor facilitates comparisons across pre-crisis, during-crisis, and post-crisis conditions, offering valuable insights into health developments throughout the pandemic. 

Recent longitudinal studies indicate an increase in mental health problems especially in high income countries [48,51,52]. This increase was also observed in the Netherlands [53]. It is recommended to consider such contextual circumstances and long-term developments when interpreting patterns and trends in population mental health based on findings from the Integrated Health Monitor COVID-19 described in the current protocol, especially as they might complicate attempts to determine the impact of the virus infections and measures in one country or compare the impact across countries.

The monitor incorporates several extensive, representative databases offering diverse data types. The surveys provide self-reported data on health and therefore can detect slight changes in health not yet disclosed with a healthcare professional. This becomes particularly useful in the context of a pandemic in which healthcare was less accessible for a period of time. However, survey research methods have inherent limitations, such as selection and recall bias. Efforts have been made during data collection to create large, representative samples in which enough coverage per province/region is ensured and harder-to-reach populations are reached as well. Post-collection, data are weighted to form a sample representative of the Dutch population, factoring in variables like age, gender, province, and socioeconomic status. However, the possibility of selection bias can never be fully eliminated with survey research. To mitigate recall bias, questions are carefully formulated, often aligned with validated instruments, prompting respondents to reflect on a concise timeframe (e.g., three months).

The general practice registry data provide objective, clinically diagnosed data, systematically recorded by each professionally trained general practitioner. This approach of using registry data is not affected by selection or recall bias and has no impact on the patient. However, it is important to note that individuals who do experience health problems but refrain from seeking healthcare are not accounted for in the data. This introduces a potential for bias, as certain populations may exhibit a tendency to avoid healthcare more than others. By incorporating both self-reported data and registry data in the monitor, these distinct data collection methods complement each other.

Whereas much of the existing health disaster research predominantly focuses on short, acute crises, the Integrated Health Monitor COVID-19 presents a unique opportunity to study a prolonged, creeping crisis experienced by the entire population. Previous studies on the effects of the COVID-19 pandemic have largely relied on cross-sectional questionnaires without pre-crisis measures, as highlighted in the review by Lin et al. [54]. In disaster health research, the unavailability of pre-event data is a common challenge, making it difficult to distinguish which effects are related to the crisis and which are not, and information is only provided about a limited time frame in relation to the event. Consequently, one of the advantages of the Integrated Health Monitor COVID-19 is that it upscales from existing structures, allowing for comparison over time and enabling the comparison of pre-crisis, during-crisis, and possibly post-crisis data. As a result, the monitor provides insight into the health status of Dutch citizens before the pandemic reached the Netherlands, as well as during different periods in the pandemic, with varying amounts of protective measures taken. This approach enables tracking of well-being over time and allows for the consideration of preexisting (mental) health conditions. These preexisting factors are typically strong predictors of experiencing negative effects during a disaster or crisis. Given that the monitor will continue until the end of 2025, it will also capture information beyond the peak of the pandemic. This extended timeframe contributes to a more comprehensive understanding of the trajectories, duration, and course of symptoms and health complaints, which allows insight into resilience trajectories within the population following a widespread, creeping crisis.

Another strength lies in the iterative approach adopted in the research. By incorporating insights from previous data collection moments to enhance subsequent rounds of data collection and analysis, the most useful and relevant information can be gathered. This involves taking the evolving knowledge needs from practice and policy into account. An illustrative example is the annual execution of literature reviews, guiding the selection of variables and outcomes for other components of the monitor. Given that the monitor outlined in this protocol paper is a complex undertaking in an ever-changing society, decisions and adjustments made during its duration are influenced by societal developments. Therefore, while this protocol outlines the principal components and original plans for the monitor, the specifics may undergo changes to stay pertinent to social development.

## Data Availability

The datasets generated during and/or analyzed during the current study are not publicly available. Funding organization ZonMw intends to make the results of publicly funded research available online to everyone. Publications must be Open Access which means they are publicly available online. ZonMw stimulates the reuse of underlying data; questions about access can be directed to Elske Marra (elske.marra@rivm.nl).

## Supplementary Materials

The following supporting information can be downloaded at: https://www.mdpi.com/article/10.3390/mps6060117/s1, Table S1: Included outcome parameters in the surveys (GGD Public Health Monitor and/or Panel Surveys); Table S2: Included ICPC-codes short-cycle general practitioners registry analysis; Table S3: Included variables in long-cycle general practitioners registry analysis.
