# Supplementary material for: The Integrated Health Monitor COVID-19: A Protocol for a Comprehensive Assessment of the Short- and Long-Term Health Impact of the Pandemic in the Netherlands
Source: Methods Protoc. 2023 Dec 2;6(6):117. doi: 10.3390/mps6060117 (PMC10745633; doi:10.3390/mps6060117)
Supplement: Supplementary file 1 [file mps-06-00117-s001.zip › mps-2671299-supplementary.pdf]

Supplementary material: outcome parameters

Table S1: Included outcome parameters in the surveys (GGD Health Monitor and/or Panel Surveys)

Perceived Health
Happiness
Psychosocial health/psychological symptoms
Suicidal thoughts
Social support
Social interaction
Resilience
Loneliness
Outlook on the future
Stress
Events experienced during the pandemic
PTSD symptoms
Lifestyle
Substance use: tobacco, alcohol, other drugs
Impact of the pandemic: negative, positive, in what ways?
Physical symptoms: non-specific physical symptoms, long-covid symptoms
Delayed care
Need for care and support

When possible, these concepts are measured with validated instruments.

Table S2: Included ICPC-codes short-cycle general practitioners registry analysis

**Complaint/condition**	**ICPC code(s)**
Fatigue*	A04
Nausea	D09
Pain or pressure in the chest*	K01 + K02 + K03
Heart palpitations*	K04
Muscle soreness*	L18
Headache*	N01 + N02
Dizziness/lightheadedness	N17
Feeling anxious/nervous/tense	P01
Acute stress reaction	P02
Feeling depressed/down	P03
Sleep disturbance	P06
Memory disturbance*	P20
Respiratory problems*	R02 + R03 + R04 + R29
Disturbance of taste and smell	N16

* Symptoms possibly related to long-covid

Table S3: Included variables in long-cycle general practitioners registry analysis

**Complaints/condition**	**ICPC code(s)**
Respiratory problems	R02 + R03 + R04 + R29
Mental symptoms	P01 + P02 + P03
Coughing	R05
Headache	N01 + N02
Fatigue	A04
Nausea	D09
Memory disturbance	P20
Dizziness	D09

Sleep disturbance	P06
Irritability/anger issues	P04
Suicidality (thoughts, attempts and completed suicides)	P77
Disturbance of taste and smell	N16
Cluster weight change	T07 + T08
Obesity	T82
Anxiety	P01 + P74
Depression	P03 + P76
Problems with education	Z07
Loneliness	Z04
Problems with the accessibility of care	Z10
Social problems	Z01 + Z03 + Z04 + Z05 + Z06 + Z07 + Z10 + Z12
Suicide	n.a. (CBS microdata does not contain ICPC codes)
**Medication prescriptions**	**ATC code**
Antidepressants	N06A
Anxiolytics	N05B
Antipsychotics	N05A
Painkillers	N02B
**Healthcare usage**	
GP practice support mental healthcare (POH-GGZ)	
Consults shorter than 5 minutes	
Consults between 5 and 20 minutes	
Consults longer than 20 minutes	
Regular house visits shorter than 20 minutes	
Regular house visits longer than 20 minutes	
**Potentially vulnerable groups**	**Definition**
People with pre-existing mental conditions	Patient registered with one of the following ICPC codes in 2019 and/or 2020: P01 P02 P03 P04 P15 P18 P19 P28 P29 P71 P72 P73 P74 P76 P77 P78 P79 P80 P98 P99
People with pre-existing chronic diseases/conditions and pre-existing physical comorbidities	Patient registered with one of the following ICPC codes in 2019 and/or 2020: T90 R83 R88 R89 R91 R95 R96 A79, B72, B73, B74, D74, D75, D76, D77, F74.01, H75.01, K72.01, L71.01, N74, R84, R85, S77, T71, U75, U76, U77, W72, X75, X76, X77, Y77, Y78, K28, K73, K74, K75, K76, K77, K78, K79, K80, K81, K82, K83, K84, T82 (HIV + immunodeficiency), B90 T99
People experiencing social problems	Patient registered with one of the following ICPC codes in 2019 and/or 2020: Z04 Z05 Z07 Z10 Z12 Z16 Z20 Z21 Z24 Z25 (NZR data)
Children and young adults	0-25 years old
Gender	Women compared to men
People with a low socioeconomic status	Definition used by CBS: those under the low-income limit. https://www.cbs.nl/en-gb/our-services/methods/definitions/low-income
People with a migration background	Definition used by CBS: https://www.cbs.nl/en-gb/our-services/methods/definitions/migration-background
